# Organic electrode coatings for next-generation neural interfaces

**DOI:** 10.3389/fneng.2014.00015

**Published:** 2014-05-27

**Authors:** Ulises A. Aregueta-Robles, Andrew J. Woolley, Laura A. Poole-Warren, Nigel H. Lovell, Rylie A. Green

**Affiliations:** ^1^Graduate School of Biomedical Engineering, University of New South WalesSydney, NSW, Australia; ^2^School of Medicine, University of Western SydneySydney, NSW, Australia

**Keywords:** coatings, carbon nanotubes, conductive polymers, hydrogels, living electrodes, material properties

## Abstract

Traditional neuronal interfaces utilize metallic electrodes which in recent years have reached a plateau in terms of the ability to provide safe stimulation at high resolution or rather with high densities of microelectrodes with improved spatial selectivity. To achieve higher resolution it has become clear that reducing the size of electrodes is required to enable higher electrode counts from the implant device. The limitations of interfacing electrodes including low charge injection limits, mechanical mismatch and foreign body response can be addressed through the use of organic electrode coatings which typically provide a softer, more roughened surface to enable both improved charge transfer and lower mechanical mismatch with neural tissue. Coating electrodes with conductive polymers or carbon nanotubes offers a substantial increase in charge transfer area compared to conventional platinum electrodes. These organic conductors provide safe electrical stimulation of tissue while avoiding undesirable chemical reactions and cell damage. However, the mechanical properties of conductive polymers are not ideal, as they are quite brittle. Hydrogel polymers present a versatile coating option for electrodes as they can be chemically modified to provide a soft and conductive scaffold. However, the *in vivo* chronic inflammatory response of these conductive hydrogels remains unknown. A more recent approach proposes tissue engineering the electrode interface through the use of encapsulated neurons within hydrogel coatings. This approach may provide a method for activating tissue at the cellular scale, however, several technological challenges must be addressed to demonstrate feasibility of this innovative idea. The review focuses on the various organic coatings which have been investigated to improve neural interface electrodes.

## INTRODUCTION

Neurological injuries and disorders affect up to a billion people worldwide and this number is estimated to increase considerably as life expectancy continues to rise (World Health Organization, 2006). Neuroprosthetic intervention is an increasingly popular method for alleviating symptoms or returning function to patients suffering from these disorders. Despite the impressive results of some electrical therapies, such as auditory implants (McCreery, 2008; Shannon, 2012), deep brain stimulators (DBS; Andrade et al., 2006; Perlmutter and Mink, 2006; Lozano and Lipsman, 2013), functional electrical stimulation (FES) of the spinal cord (Collinger et al., 2013) and vision prostheses (Shepherd et al., 2013), considerable improvement in device technology is required to enable greater control of physiological outcomes (Normann, 2007). Current state-of-the-art neuroprostheses generate an electrical field in the target tissue using metallic electrodes to elicit or suppress neuronal action potentials (Perlmutter and Mink, 2006; Li and Mogul, 2007; Normann, 2007; Wilson and Dorman, 2008; Collinger et al., 2013; Shepherd et al., 2013). Many such devices also use the same metallic electrodes to record neural responses (Normann, 2007). Most metallic electrodes inject charge through the generation of electrons at the electrode surface, however in physiological systems charge is carried by electrolytes (ions). At the electrode–electrolyte interface, charge must be transferred from electrons to ions by either Faradic (electrochemical reactions) or capacitive (double-layer charging) mechanisms which are dependent on the material selected for the electrode (Merrill et al., 2005; Cogan, 2008). Typically platinum (Pt), gold and platinum-iridium (Pt-Ir) are used for fabricating biomedical electrodes (Brummer et al., 1983; Geddes and Roeder, 2003). Pt has historically been considered the preferred metal used for electrodes in neuroprostheses (Brummer et al., 1983; Cogan, 2008), with cochlear implants, DBS and retinal implants all using Pt for neural interfacing. This is due to the electrochemical stability and corrosion resistance of Pt (White and Gross, 1974) which has been demonstrated to have limited reactivity to biological environments compared to other metals (Merrill et al., 2005; Polikov et al., 2005). However, while technological advances have driven the miniaturization of electronics leading to smaller implant devices (Nakayama and Matsuda, 1995; Schuettler et al., 2005; Cheung, 2007; Wester et al., 2009), aspects of Pt electrical, mechanical, and biological performance remain as limiting factors which prevent the application of high-density microelectrode arrays for neural interfacing.

For every electrode there is an intrinsic charge injection limit, restricting the voltage that can be safely generated at the electrode’s surface. Once this voltage is breached, purely capacitive charge transfer can no longer be maintained and irreversible faradaic reactions occur. Beyond this electrochemical limit, also known as the water window, irreversible electrolysis of water can result in tissue damage, electrode dissolution, pH changes and production of unwanted chemical species (McCreery et al., 1997; Zhong et al., 2001; Green et al., 2008a; Poole-Warren et al., 2010; Niina et al., 2011). Additionally, as an electrode is reduced in size the charge it must pass per unit area increases, directly increasing the voltage on the electrode and hence reducing the total charge which can be safely delivered. It has been shown that to stimulate light percepts by electrical stimulation of the retina in visually impaired patients, a charge density between 48 and 357 μC/cm^2^ is required (Humayun et al., 2003; Mahadevappa et al., 2005), but the electrochemical injection limit of Pt has been reported as ranging from 20 to 150 μC/cm^2^ (Rose and Robblee, 1990; Green et al., 2012c). This small range of overlap means that bare metal electrodes cannot be safely reduced in size and still maintain safe charge injection at a therapeutic level. With the increasing pressure to reduce the size of electrodes, driven by the need to make smaller but higher resolution implants, Pt electrical properties have become a challenging issue (Green et al., 2012c; Shepherd et al., 2013). New electrode geometries, materials, or coatings must be used to increase the charge transfer surface area such that the safety limits are preserved. Surface modifications, through electrode roughening or coating, have been reported to have great potential for increasing the charge injection capacity of microelectrodes, as detailed later in this review (Cogan et al., 2005; Schuettler et al., 2005; Abidian et al., 2010; Green et al., 2012b, c, 2013a).

Mechanically, Pt is significantly stiffer than the neural tissue with which it interfaces (Green et al., 2012b). The elastic modulus of Pt is about 164 GPa (Merker et al., 2001), but most neural tissue has a modulus of less than 100 kPa (Lacour et al., 2010). This mechanical disparity can exacerbate the chronic inflammatory response at the implant site, as the shear between a stiff electrode and the soft neural tissue continues to incite inflammation during tissue movement and device micromotion (Rousche et al., 2001; Leach et al., 2010). More flexible device designs move with the tissue, which can reduce damage at the neural interface (Richter et al., 2013), but require tethering which creates damage in adjacent tissue sites (Tyler and Durand, 2002). Electrode coatings, in particular polymeric films which utilize conductive polymers or hydrogels, have been shown to impart a softer electrode interface, around 1 MPa (Yang and Martin, 2006; Green et al., 2012b) and it is expected that these coatings can be used to dampen or mediate the mechanical difference between a metal electrode and the tissue with which it interfaces.

Chronic biological responses to metal electrodes have been reported to challenge the maintenance of an effective neural interface (Turner et al., 1999; Biran et al., 2005; Barrese et al., 2013). The implantation and chronic presence of a neural interfacing device in the central nervous system (CNS) induces a cascade of biological processes which can ultimately isolate the electrode and gradually decrease device performance. Reports and reviews on implantation trauma have detailed the cellular and molecular interactions involved in the acute inflammatory response which primarily includes immune cell activation and migration, and local ischemia (Fitch and Silver, 2008; Zhong and Bellamkonda, 2008; Whitney et al., 2009). The biological environment is further altered as the ongoing inflammatory response produces reactive astrocytosis in the damaged area (Banati et al., 1993; Fitch and Silver, 1997; Babcock et al., 2003). Over time, layers of activated microglia, invading macrophages, reactive astrocytes and migrating meningeal fibroblasts can encapsulate CNS implants (Turner et al., 1999; Cui et al., 2003). Extra layers of non-excitable cells will increase the neural interface impedance. For recording electrodes this reduces the possibility of recording and localizing single unit activity due to a diminished signal to noise ratio (SNR). As Pt is a relatively stable material which has limited interaction with the biological environment, immune cells are prevented from dissolving the electrodes. However, a persistent effort to disintegrate metal sites yields a constant environment of cytotoxic factors that may contribute to migration away from and cell death near the electrodes, including the target neurons (Weldon et al., 1998). Furthermore, Pt and other metallic electrodes are typically produced with smooth surfaces which do not encourage neural tissue integration, as a result immune cells can access the gap between the electrode and target cells. Several detailed studies have described the chronic biological response to implantable electrodes in animal models (Hascup et al., 2009; Ward et al., 2009; Leach et al., 2010; Woolley et al., 2011).

**FIGURE 1:**
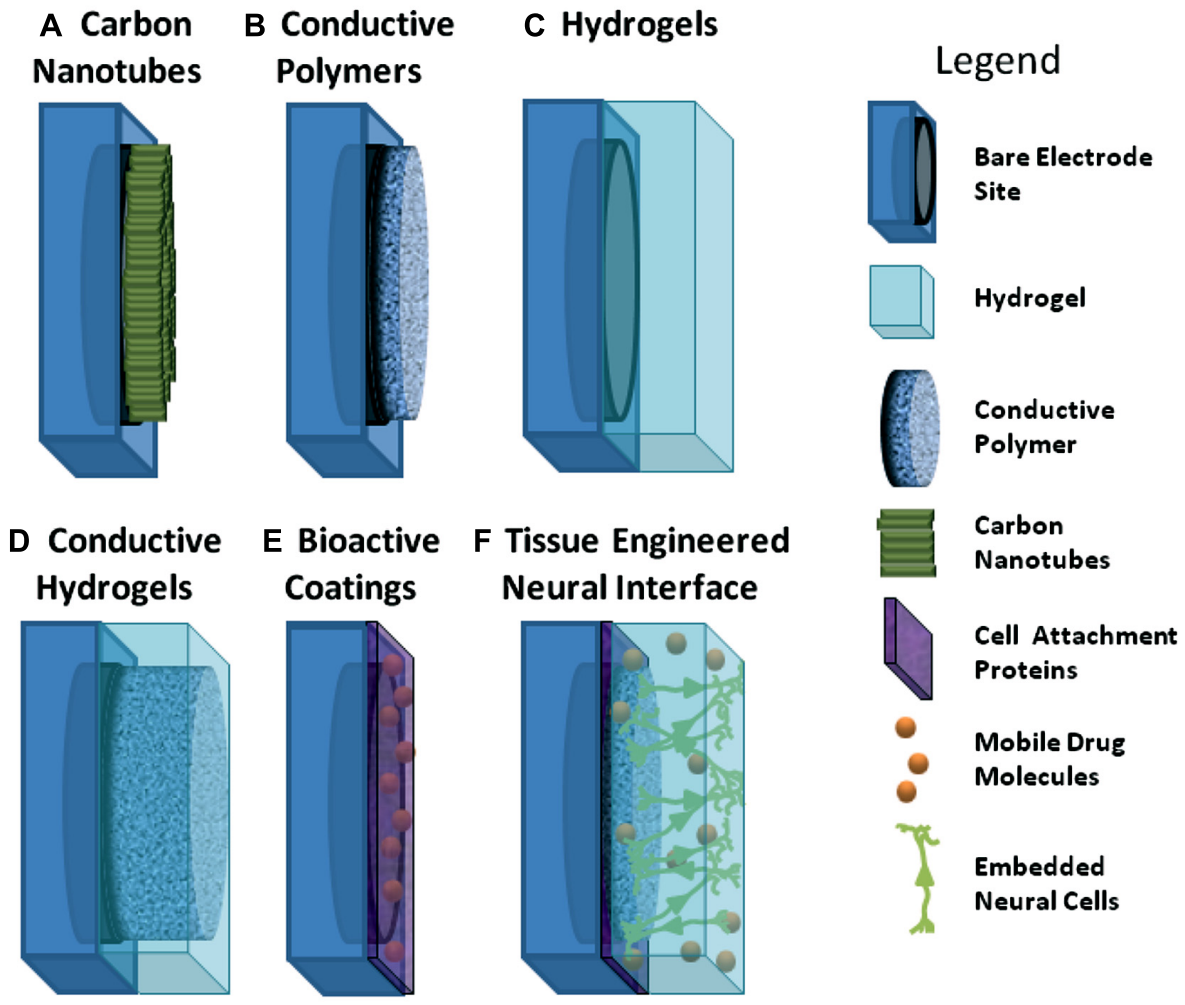
**Schematic of coating approaches used for addressing the limitations of metallic electrodes.** **(A)** aligned carbon nanotubes on metallic electrodes; **(B)** conductive polymers electrodeposited on metallic electrodes; **(C)** hydrogels polymerised to coat electrode site and device; **(D)** interpenetrating network of conductive polymer grown through hydrogel coating to form conductive hydrogel over electrode sites; **(E)** electrode site coated with biologically active molecules; **(F)** schematic of ideal tissue engineered interface incorporating combined coating approaches of conductive polymers, hydrogels and attachment factors with neural cells.

In the literature, the limitations associated with Pt electrode performance have been addressed through several varied approaches, which include passivation and surface texturing of electrodes to reduce impedance and enhance tissue integration (Cogan et al., 2005; Schuettler, 2007; Abidian et al., 2010; Green et al., 2012a, b). However, it is through the development of new coating technologies that improvement can be made more widely across the electrical, mechanical and biological properties of electrodes. In particular the use of carbon nanotubes (CNTs), CPs, hydrogels and conductive hydrogels (CHs), depicted in **Figure 1**, have shown that tailored approaches can be used to create multi-functional electrode arrays which not only improve the electrode material properties, but also provide biomolecules to aid in the establishment of a chronically stable neural interface. While substantial research has been conducted on CNTs, CPs, hydrogels and composite polymers, it is important to understand both the advantages and limitations of these materials and how they impact on the biological environment. Furthermore, as greater demands on electrode technologies drive the development of next-generation bionic devices, it is proposed that tissue engineered electrodes, such as that shown in **Figure 1** (bottom, right) may provide an avenue for directly interfacing with neural cells through synaptic communication. This review highlights the materials and emerging technologies that address some of the issues related to conventional smooth metallic electrodes including enhancing charge transfer, tissue integration and reducing mechanical mismatch. Furthermore it proposes an innovative approach to creating electrodes which use neural cells embedded within the electrode surface for a more natural approach to cell activation which may reduce scar tissue formation and aid in the establishment of a stable neural interface.

## COATINGS FOR NEURAL INTERFACES

A common objective of modifying an electrode surface is to impart roughening or rather an increased electrochemical surface area. Several roughened morphologies have been shown to enhance charge transfer to within safe stimulation limits (Rose and Robblee, 1990; Cogan et al., 2007; Green et al., 2013b) as well as providing a surface to improve neuronal attachment (Hallab et al., 2001; Fan et al., 2002). Roughening the surface can be achieved by altering the existing metallic surface or through coating with an alternate material. While direct modification of the metallic surface can impart roughness without significantly altering the electrode chemistry (Green et al., 2010c), the benefits are limited, in that the material stiffness and chemical compatibility with the biological system remains unchanged. Electrodes can be coated through a variety of methods including electrochemical deposition (Cui et al., 2003; Geddes and Roeder, 2003; Green et al., 2008a; Abidian et al., 2010; Hassler et al., 2011), physical vapor deposition (Geddes and Roeder, 2003; Gabay et al., 2007; Shoval et al., 2009; Chen et al., 2011) such as sputtering (Geddes and Roeder, 2003) or evaporation, spin-coating (Green et al., 2010c; Lacour et al., 2010) or dip-coating (Schuettler et al., 2005) from solutions which require curing (Fieberg and Reis, 2002; Chen et al., 2011; Rao et al., 2012) or cross-linking (Guimard et al., 2007). The method employed depends strongly on the type of material which is required for the coating. Electrochemical deposition is used to apply a material directly to an electrode site (Cui et al., 2001b; Bartlett et al., 2002; LaVan et al., 2003), but other methods coat the entire construct and often require post-processing to ensure the coating is applied only to the required areas of the electrode array (Kim et al., 2008; Abidian and Martin, 2009). Both organic coatings such as CNTs, CPs, and CHs, and inorganic coatings including Pt-Black, titanium nitride (TiN) and iridium oxide (IrOx) have been used to impart increased charge injection capacity to metallic electrodes (Cogan et al., 2005; Schuettler, 2007; Abidian et al., 2010; Green et al., 2012a, b). However, the organic coatings hold a significant benefit over the inorganic coatings as they can be easily modified to include functional molecules to influence the biological response. Organic polymers based on hydrogels present an alternate coating option. While hydrogels do not inherently impart a roughened surface area, they have been shown to provide mechanical and biological benefits to electrodes which, in turn improve the chronic electrical performance. Additionally, hydrogels have formed the basis of several composite coatings which utilize the combination of a conductive component embedded in a non-conductive polymer matrix.

### CARBON NANOTUBES

Carbon nanotubes have remarkable mechanical and electrical properties that exhibit noted interaction with neural tissue. CNTs are cylinders formed from seamless sheets of graphene with a wall thickness of 1 atom. Single walled CNTs (SWCNTs) as the name suggests have only one sheet creating a single cylinder, but multi-walled CNTs (MWCNTs) have multiple concentric cylinders of graphene. Some of the methods used to apply CNTs to electrodes are chemical vapor deposition (CVD) (Heim et al., 2012), immersion drying (Haghighi and Bozorgzadeh, 2011) and electrodeposition (Yang et al., 2010; Suzuki et al., 2013). CVD presents some drawbacks as it yields secondary toxic chemicals that require further purification (De Volder et al., 2013), also this technique requires higher temperatures process and this limits the possible materials that can be coated. CNT coatings produced by immersion are limited in durability, as the nanotubes are not bonded to surface. Electrodeposition is considered a simpler method to coat films with controllable thickness yielding a mechanically stable coating (Yang et al., 2010). It is worth noting that CNTs do not simply adhere to metallic substrates and as a result must be chemically modified or embedded within a polymer matrix to remain adhered to an electrode. As such all of the coatings described are composites of CNTs.

Perhaps one of the most important properties of CNTs is their ability to enhance electrical properties of metallic electrodes. The addition of CNTs to both insulating and conductive materials results in electrodes with higher charge storage capacity (CSC) and lower impedance (Green et al., 2008b; Xiao et al., 2012; David-Pur et al., 2013). By increasing the thickness of a coating containing CNTs the CSC has been reported to be as high as 70 mC/cm^2^ (Luo et al., 2011b), although in this study the CNTs were embedded in a CP which likely contributed to the high CSC. It has also been shown that the presence of CNTs in a CP composite can stabilize the electrochemical properties of the coating (Green et al., 2008b). Similarly, several studies on electrodes coated with CNTs report a substantial increase in SNR for recording electrodes (Gabay et al., 2007; Yu et al., 2007; Keefer et al., 2008; Lin et al., 2009; Shoval et al., 2009; Hsu et al., 2010; Suzuki et al., 2013) which is thought to be predominantly due to the low impedance CNTs impart to electrodes (Keefer et al., 2008). In particular, (Shoval et al., 2009) presents an evident increase in SNR when recording neuronal activity from retina tissue. In this work CNT coatings increased the ability to record voltage spikes, increasing the SNR by up to three times in comparison with titanium nitride electrodes.

The low volume, high surface area of nanotubes means that they can dramatically increase the charge transfer area of an electrode. Their nanoscale features which enable them to penetrate cellular membranes, are also expected to enhance electrical performance by promoting a more intimate interaction with tissue. (Suzuki et al., 2013) detected action potentials and field postsynaptic potentials employing a multi-electrode array (MEA) coated with planar CNTs. In stimulation electrodes, a low impedance and decreased activation threshold have been achieved by several groups who have embedded CNTs in flexible polymer substrates including parylene-C, poly(dimethyl siloxane) (PDMS) and the CP polypyrrole (Nguyen-Vu et al., 2006; Wang et al., 2006; David-Pur et al., 2013). The safe charge injection limit of CNT containing coatings have been reported to be between 1.6 and 2.5 mC/cm^2^ (Wang et al., 2006; Luo et al., 2011b). This is more than 10 times the charge injection reported for Pt.

**FIGURE 2:**
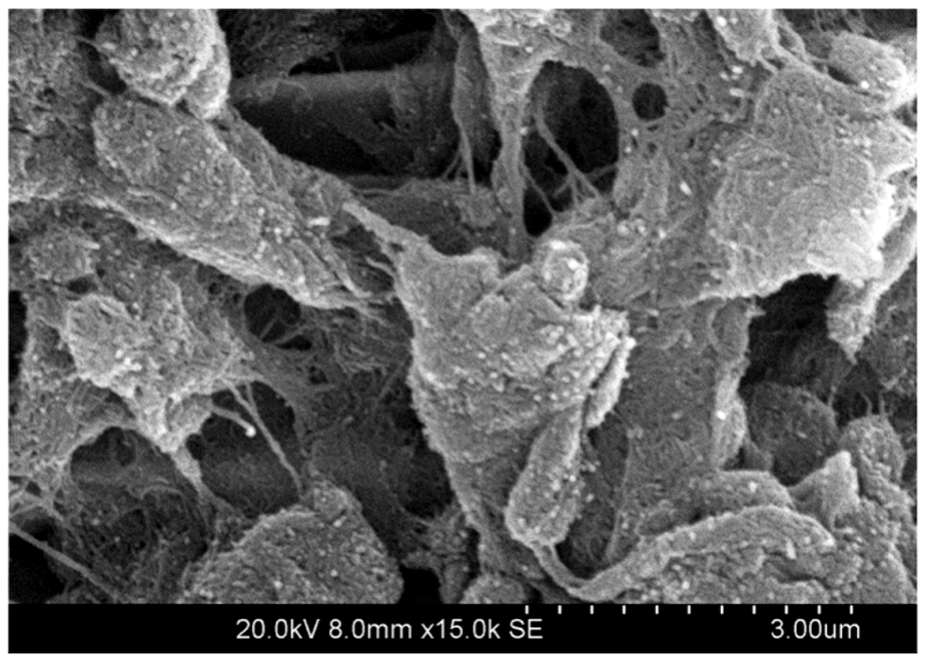
**Scanning electron microscope (SEM) image of multi-walled carbon nanotubes (MWNTs) coating a platinum disk electrode, demonstrate that CNTs produce fibrillar surface structures, imparting a high charge transfer area to the typically flat electrode.** The platinum disk is not visible, as the entire substrate is covered with CNT bundles. Image produced at 15,000× magnification.

Carbon nanotubes are very stiff materials with a Young modulus of 1.25TPa (Krishnan et al., 1998). This is a significant drawback when interfacing with neural tissue where mechanical mismatch between the device and cells is one of the main factors contributing to chronic inflammation. However, it is arguable that the nanoscale dimensions of these materials minimize the impact of shear stress at the cellular interface. In fact the ability for nanotubes to penetrate cells has been well detailed (Kagan et al., 2006; Tian et al., 2006; Gilmour et al., 2013). Additionally, nanotube morphology has promising properties for neural tissue engineering, with most coatings having a fibrillar surface, as shown in **Figure 2**. This morphology presents the cells with a nanometric roughness that is thought to create a more intimate cell-electrode interface (Keefer et al., 2008; Seidlits et al., 2008) suitable for cellular attachment.

Despite the promising electrical and physical properties of CNTs their biocompatibility is a subject of considerable discussion. Being small and biologically inert provides CNTs with the ability to infiltrate tissues without being identified by the immune system (Seidlits et al., 2008). While these nanoscale structures are less likely to be identified by reactive astrocytes and consequently may minimize scar tissue formation, (Kagan et al., 2006), it has been suggested this may represent a hazard as internalized CNTs can damage the intracellular bodies including the nuclei of cells. Additionally, it has been shown that high concentrations of CNTs induce cytotoxicity, either as un-functionalised SWCNTs or as composites with other polymers. Cell death in a dose dependent manner has been reported for lymphocytes (Bottini et al., 2006) and fibroblasts (Tian et al., 2006). Likewise, Webster et al. (2004) created CNT composites with polycarbonate urethane and their results showed that the toxicity of the composite increased whenever the proportion of the CNTs in the composite was 10% or more. It is clear that the biocompatibility of CNTs will remain a controversial subject, but coatings which can constrain the CNTs while utilizing their impressive electrical conductivity have significant potential in the future of neural interfaces.

### CONDUCTIVE POLYMERS

**FIGURE 3:**
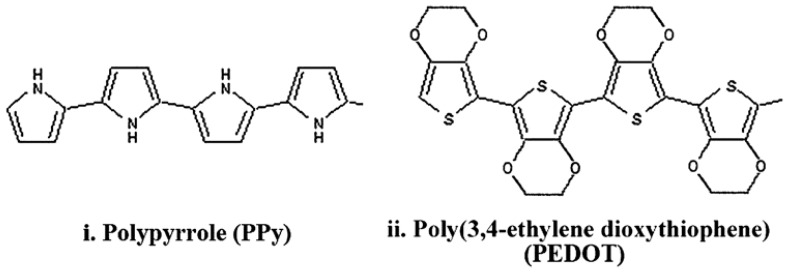
**Structure of PPy and PEDOT with alternating single and double bonds along the backbone which impart conductivity (Green et al., 2008a**).

CPs are synthesized from chains of chemical compounds that present alternating double and single bonds in their structure (Guimard et al., 2007). This structure, known as a conjugated system, confers the conductive property to the polymer. When doped with an appropriately charged ion to stabilize the backbone, high conductivity can be obtained (Guimard et al., 2007). CPs can inject both electronic and ionic charge (Cogan, 2008) and have been used to both stimulate nerve tissue and record neuronal activity (Kim and Romero-Ortega, 2012). Among several conductive polymers, polypyrrole (PPy) (Cui et al., 2003; Kim et al., 2004; George et al., 2005; Stauffer and Cui, 2006; Green et al., 2008a) and poly(ethylene dioxythiophene) (PEDOT; Cui and Martin, 2003; Xiao et al., 2004; Yang et al., 2005; Ludwig et al., 2006, 2011; Green et al., 2008a), shown in **Figure 3** have been extensively used to coat neuroprosthetic electrode sites. Other CPs which have been investigated to a lesser extent include polyterthiophene (Stevenson et al., 2010), polyaniline (Huang et al., 2004; Bidez et al., 2006) and various modifications of PEDOT such as methoxy-PEDOT (PEDOT-MeOH), carboxylic acid modified PEDOT (PEDOT-COOH) and propylenedioxythiophene (Pro-DOT) (Martin and Feldman, 2012). PEDOT is considered one of the most promising CPs due its electrical and chemical stability in an oxygenated, hydrated environment (Cui and Martin, 2003; Guimard et al., 2007; Green et al., 2013b).

The fabrication method and the choice of CP components are critical to determining the resulting mechanical, chemical and electrical performance (Baek et al., 2013a). CPs can be fabricated by either chemical or electrochemical methods. While chemical synthesis enables the development of complex and highly ordered structures, the difficulties in applying these materials to metallic electrodes have largely hindered their investigation and application to neuroprosthetics. Additionally, chemically synthesized CPs require post-process doping to reach similar conductivities to electrochemically polymerized CPs. Electrodeposition is the most common method used to fabricate CP coatings on electrodes. This method enables direct formation of the polymer on the electrode site and by varying the time over which electrodeposition occurs, the thickness and roughness of the CP can be controlled (Fonner et al., 2008; Baek et al., 2013a). The dopant ion is included in the monomer electrolyte solution and is directly incorporated within the CP. Varying the dopant type and concentration also impacts on the CP electrical (Guimard et al., 2007) and morphological properties (Green et al., 2012a). While cations can be used to dope CPs, anions are almost exclusively used for electrodeposited CPs which are polymerized by oxidation producing a positively charge backbone, requiring a negatively charged dopant (Guimard et al., 2007). The most effective dopants for producing CPs with high charge transfer area and stable electrochemical properties are ions with sulfonate moieties, although phosphates and perchlorates have been successfully used to fabricate CP coatings with high charge transfer capacity. Common dopants which have been extensively investigated have included poly(styrene sulfonate) (PSS), paratoluene sulphonate (pTS), dexamethasone phosphate (Dex-P), and perchlorate (ClO_4_).

**FIGURE 4:**
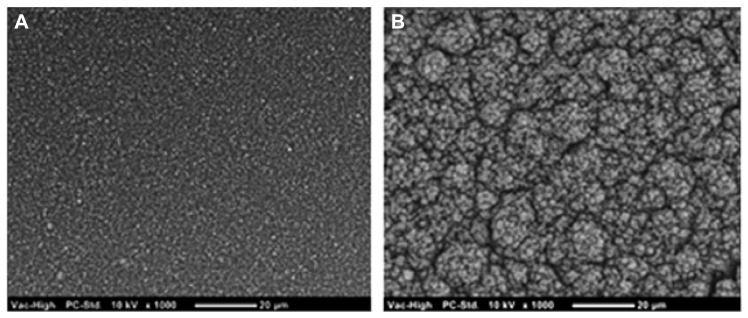
**Scanning electron microscope images show that (A) PEDOT doped with PSS produces a coating which is substantially smoother than that produced from **(B)** PEDOT doped with pTS**.

CPs have been shown to improve on the electrical performance of Pt across various metrics important to electrode function, including the CSC (Cogan, 2008; Abidian et al., 2010; Green et al., 2013b), the electrochemical impedance (Kim et al., 2004, 2008; Abidian et al., 2010; Green et al., 2013b), and the charge injection limit (Kim et al., 2004, 2008; Green et al., 2012a, b). A summary of the various CPs and their electrical properties is shown in **Table 1**. In general CPs produced from PEDOT and doped with small sulfonate ions, namely pTS, have the highest charge storage capacity, lowest impedance and highest injection limit. Coating Pt electrodes with PEDOT/pTS can increase the CSC by one order of magnitude and the charge injection limit by up to two orders of magnitude (Cogan, 2008; Green et al., 2013b). Similarly, in the latter work the frequency dependant impedance is also reduced in the low frequency range, where it is proposed that capacitive double layers are the dominant mode of charge transfer (Green et al., 2013b). These improvements in electrical properties are primarily due to the increase in the charge transfer area of the electrode which occurs as a result of the intrinsic rough morphology of PEDOT. When large anions or polymers are used to dope PEDOT the surface is typically smoother which results in poorer electrical performance, such as that shown in **Table 1** for PEDOT/PSS. Small dopants enable more efficient polymerization than larger dopants, and structurally enable greater flexibility in the backbone during PEDOT formation. As a result these smaller dopants create rougher, more nodular surfaces. The comparison of PEDOT/pTS and PEDOT/PSS surface morphology is shown in **Figure 4**. Since common analytical techniques such as stylus profilometry and indentation (including scanning AFM) can damage the CP surface, there are very few papers in the literature which quantify CP surface roughness. With the recent development of non-contact optical profilometry there is now an opportunity to provide measurements of roughness which can be related to the electrical performance of CPs. Using optical profilometry (Green et al., 2013b) have reported that PEDOT/pTS coatings can increase the surface area of a smooth electrode by a factor of 2, which closely correlated to the reduction in voltage drop across Pt electrodes coated with this CP (Green et al., 2013b). The electrical stability of CP electrode coatings is also related to the chemical constituents, with literature reporting minimal changes in PEDOT/pTS following more than 2 billion pulses (Boretius et al., 2011; Green et al., 2012a). However Yamato et al. (1995) reported loss of 95% of the electroactivity of PPy/PSS following 16 h of electrical polarization. 

**Table 1:** Electrical properties of conductive polymers.

Conducting polymer	Charge storage capacity (CSC) (mC/cm^**2**^)	Impedance @ 1000 kHz (ømega/cm^**2**^)	Electrochemical charge injection limit (mC/cm^**2**^)	Reference
PEDOT/pTS	245.59^S^; 402.23^R^	2.65 × 10^3^	2.09 ± 0.2^S^; 2.01 ± 0.4^R^	Green et al. (2009),2012a
PEDOT/PSS	105.17^S^; 243.48^R^	2.03 × 10^3^	1.36 ± 0.1^S^; 1.52 ± 0.5^R^	Green et al. (2012a), Baek et al. (2013a)
PEDOT/ClO_4_	98.49^S^; 389.88^R^	2.03 × 10^3^	2.39 ± 0.4^S^; 2.09 ± 0.5^R^	Baek et al. (2013a)
PPy/PSS	186.4	256.41 × 10^6^	–	Cui et al. (2001a),b
PTT/Dex-P	~35	–	–	Stevenson et al. (2010)
PProDOT	5.8	–	–	Rosario-Canales et al. (2011)
Poly(EDOT-COOH)	–	~10	–	Luo et al. (2012)
P(PRoDOT-OH)	–	~10	–	Luo et al. (2012)

*S, coated on smooth platinum. R, coated on roughened platinum.*

Mechanically CPs are largely claimed to reduce the stiffness of the electrode interface, however, few papers in the literature actually quantify the modulus of CPs. Several reports of dry CP films based on PEDOT demonstrate that the elastic modulus varies with dopant choice, but generally are in the range of 1–3 GPa (Yang and Martin, 2006; Baek et al., 2013a). More recent papers demonstrate that when hydrated the elastic modulus of CPs, in particular PEDOT/PSS can be as low as 40 MPa (Green et al., 2012b).This is substantially lower than the elastic modulus of Pt, but still more than two orders of magnitude higher than neural tissue. While CPs may have the capacity to dampen the mechanical mismatch at the neural interface, the mechanical stability is a greater concern. Several reports claim that delamination of these coatings can occur *in vivo* (Peixoto et al., 2009; Boretius et al., 2011) and moreover the surface, in particular of the highly rough PEDOT, is brittle and friable (Collier et al., 2000; Green et al., 2010b). While CPs have the benefit of being able to be functionalized to incorporate biological components, the addition of these large molecules further diminishes the mechanical stability of these coatings. Several approaches, including chemical tethering (Labaye et al., 2002) and mechanical interlocking (Green et al., 2012a) have been used to improve the adherence of CP coatings, but polymer cohesiveness requires improvement.

Various CPs have been shown *in vitro* to have cytocompatible properties, with several cell types including neuroblastomas (Bolin et al., 2009), spiral ganglion cells (Evans et al., 2009) and pheochromocytoma (PC12) neural model cells (Schmidt et al., 1997; Green et al., 2009). In spite of the promising results, little *in vivo* evidence has been presented to support the chronic stability and the benefit of applying CPs to neural interfacing electrodes. (Wilks et al., 2011) polymerized PEDOT *in vivo* within the rodent cerebral cortex, creating a direct CP interface with the neuronal tissue. In this work (Wilks et al., 2011) reported lowered impedance values, improved recording quality of local field potentials, and a tight cloud of PEDOT penetrating into the tissue surrounding the electrode. (Cui et al., 2003) were able to efficiently record neuronal activity for 2 weeks by implanting gold electrodes coated with PPy in guinea pigs cerebral cortex. After 2 weeks the polymer underwent structural changes and scar tissue encapsulation started to decrease electrode performance. Similarly, (Ludwig et al., 2006) demonstrated that CPs improve neural recordings using a PEDOT/PSS coated Michigan probe electrode array implanted in the rat motor cortex for 6 weeks. However impedance increased over time with a respective reduction in SNR correlating with a neural density decrease near the electrodes. It was suggested that neuronal loss was due to implant trauma. Recent evidence suggests that neurons not only may die following damage during the implantation process but can also migrate away from the electrode as the neuroglia isolates the device as a result of foreign body reactions (McConnell et al., 2009).

CPs address some of the limitations associated with reducing the size of Pt electrodes, providing electrical, mechanical and biological benefits. Despite the ability of CPs to enhance electrode performance, issues remain concerning mechanical stability and inflammatory reactions in the chronic implant environment. Approaches to improve the biological performance of CPs have included the development of composite CP-hydrogels and the use of biological inclusions intended to influence the cellular response.

### HYDROGELS

The need to develop an electrode interfacing material with an elastic modulus similar to that of nerve tissue is a recurring concept (Kim et al., 2008). Hydrogels are polymer systems which have been extensively studied for tissue engineering (Lee and Mooney, 2001; Hoffman, 2002).The networks are held together by physical or chemical crosslinks and network design can incorporate a range of degradation profiles or may be non-degradable. Structure and mechanical properties of hydrogel networks can be controlled through selection of fabrication technique and chemical composition (Lacour et al., 2010). These crosslinked polymeric networks have high water contents making them an attractive platform for neural interfacing. Hydrogels are commonly fabricated from either natural materials such as collagen (Ma et al., 2004; Mao and Kisaalita, 2004; Suri and Schmidt, 2010), fibrin (Georges et al., 2006; Ahmed et al., 2008), and alginate (Novikova et al., 2006; Banerjee et al., 2009) or synthetic materials like polyvinyl alcohol (PVA) (Lu et al., 2009; Lim et al., 2012, 2013), polyethylene glycol (PEG; Drury and Mooney, 2003) and polyacrylamide (Georges et al., 2006). Synthetic polymers enable controlled hydrogel mechanical properties but lack cellular activity due to the absence of bioactive elements. On the other hand natural polymers support enhanced cellular interactions but form constructs that are less mechanically and dimensionally stable compared with synthetic hydrogels. Co-hydrogel or biosynthetic systems combine the benefits of natural and synthetic polymers and permit greater control over the polymer properties. While many hydrogel systems have been investigated for tissue engineering, only a few types have been applied to neuroprosthetic electrode arrays.

Although few hydrogels are considered conductive, most are poor insulators due to the open polymer mesh structure which swells in aqueous environments. Winter et al. applied poly(ethylene glycol)-poly(lactic acid) (PEGPLA) to multielectrode arrays and found that the electrochemical characteristics of the underlying IrOx coated electrode array were preserved (Winter et al., 2007) however, the thickness of the layer was not reported. Zhong and Bellamkonda coated electrode arrays with a nitrocellulose based hydrogel and found that impedance of the neural interface was reduced (Zhong and Bellamkonda, 2005). Hydrogel mesh structures provide minimal hindrance to the ions required for capacitive charge transfer. Furthermore, some hydrogels have a strongly charged backbone which encourages ion sequestering (Hoffman, 2002). These ions provide a ready source of charge for formation of a capacitive double layer at an underlying metallic electrode interface. However, it is also observed that when a hydrogel coating swells *in vivo*, it can push the target neural tissue further away from the electrode (Kim et al., 2010), necessitating higher charge delivery to activate the neurons.

By developing hydrogels with a mechanical modulus within the range of nerve tissue, less than 100 kPa (Lacour et al., 2010), it has been suggested that hydrogel coatings can reduce stress related to micromotion of stiff materials (Kim et al., 2010). The compression modulus of hydrogels is highly variable and strongly dependant on the type of polymer used for coating, a summary of hydrogel moduli is presented in **Table 2**. In a study by Baek, hydrophilic brushes fabricated from polyhydroxyethylmethacrylate (pHEMA) were applied to gold electrodes and found to reduce the modulus from 70 GPa to 143 MPa (Baek et al., 2013b). In a similar study by Green et al. (2012b), Pt was coated with the biosynthetic co-hydrogel polyvinyl alcohol-heparin (PVA-Hep) and the electrode modulus was reduced to near 100 kPa. Several studies on hydrogel chemistry and structure have shown that the mechanical properties of synthetic polymers can be controlled through varying the length of the polymer backbone, the number of crosslinks and the percentage polymer composition relative to the water content (LeRoux et al., 1999; Normand et al., 2000; Almany and Seliktar, 2005; Nilasaroya et al., 2008). However, a material that is as soft as the tissue presents difficulties during implantation (Kim et al., 2008; Richter et al., 2013). In an effort to address the latter issue, recent research has investigated novel hydrogel materials which have switchable properties when exposed to different environmental conditions. Capadona et al., developed a cellulose-whisker nanocomposite that softens when the temperature is increased from 25 to 37°C. It was shown that the initial stiffness of the arrays produced with this material were 4.2 GPa which was reduced to 1.6 MPa in 10 min following immersion in physiological fluid at 37°C (Capadona et al., 2008). It is conceivable that through choice of polymer and control of the hydrogel structural characteristics, the mechanical modulus of an electrode can be tailored for the neural interface.

**Table 2:** Mechanical moduli of various hydrogels.

Hydrogel	Type (synthetic, natural, or biosynthetic)	Modulus range (kPa)	Reference
Polyvinyl alcohol (PVA)	Synthetic	2560–7360^*^	Baker et al. (2012)
Polyethylene glycol (PEG)	Synthetic		
Poly(2-hydroxyethyl methacrylate) (pHEMA)	Synthetic	440 ± 10	Jhaveri et al. (2008)
Poly(lactic-co-glycolic acid)	Synthetic	752 ± 54	Vaquette et al. (2010)
Collagen	Natural	0.5–12	Raub et al. (2010)
Agarose	Natural	1.5–2580^**^	Letherby and Young (1981), Normand et al. (2000), Aymard et al. (2001), Ahearne et al. (2005)
Alginate	Natural	1–8^**^	LeRoux et al. (1999)
PVA-heparin	Biosynthetic	60–68^**^	Nilasaroya et al. (2008)
PEG-Fibrinogen	Biosynthetic	0.01–2^**^	Almany and Seliktar (2005)

* Values change depending on confined compression **Values are proportional to the percent polymer composition.

Cell interactions with hydrogels, like the mechanical properties, depend on the polymer choice. Synthetic hydrogels generally have poor cell interactions which have made them useful as antifouling coatings for implants (Telford et al., 2010; Liu et al., 2012). This property may be of use in neural prosthetics where protein deposition, which increases the impedance of electrodes, can be prevented (Li et al., 2005; Green et al., 2010a). However, a significant benefit of synthetic hydrogel systems is that they can be modified to incorporate biological molecules. The ability to functionalize hydrogels has been extensively explored (Hoffman, 2002; Willerth and Sakiyama-Elbert, 2007) and they can be modified to include covalently bound bioactive molecules, to bind and release growth factors (Nilasaroya et al., 2008), or to degrade over time to allow controlled drug release (Anseth et al., 2002). (Zhong et al., 2001) demonstrated that natural hydrogels of collagen promoted cortical nerve cell attachment. Lim et al. have further shown that very small amounts of the collagen derivative gelatin, incorporated at 0.01–1 wt% within a synthetic PVA hydrogel can promote attachment of various cell types including neuronal cells (Lim et al., 2013). Further, Winter et al. used degradable PEGPLA to successfully deliver nerve growth factor (NGF) and promote the outgrowth of neuronal processes from PC12 cells (Winter et al., 2007). Several studies have examined release of drugs from hydrogel coatings (Zhong and Bellamkonda, 2005; Winter et al., 2007) and the issues related to drug delivery are discussed below in relation to bioactive coatings. Ultimately, further studies are required to develop a hydrogel which can soften device interfaces while limiting the gap between the electrode and tissue.

As hydrogels do not substantially improve the electrical properties of the underlying material, they can only go part of the way to addressing the limitations of metallic electrodes. However, there is a wealth of literature on the design, fabrication and characterisation of hydrogel systems for alternate biomedical applications (Lee and Mooney, 2001; Hoffman, 2002; Yu and Bellamkonda, 2003; Kurisawa et al., 2005; Lee et al., 2008; Nicodemus and Bryant, 2008; Winter et al., 2008; Zuidema et al., 2011; Abidian et al., 2012). It is expected that through consideration of this body of work a systematic assessment of various hydrogel chemistries and structures will allow selection of appropriate polymers for improving the mechanical softness and biological interaction of neural electrode arrays. It is proposed, however, that such a hydrogel will require additional modification to incorporate conductive components to ultimately improve charge transfer and facilitate the development of high density microelectrode arrays.

### CONDUCTIVE HYDROGELS

Blending hydrogels with conductive components such as CNTs or CPs has provided an avenue for maintaining the desired electrical characteristics of a coated electrode while imparting reduced stiffness (Green et al., 2012b; Xiao et al., 2012). The hydrogel component also presents the opportunity to incorporate bioactive molecules at higher concentrations than in CNT or CP films alone. Since CNTs usually require a polymer matrix to constrain them within the electrode coating, composites based on CNTs have been discussed above. CP-hydrogel hybrids (known as conductive hydrogels) are a more recent development which address the limitations of homogenous CPs (Green et al., 2010a, 2012a). As the CP component is fabricated within the hydrogel matrix, the friable particulates are encapsulated, improving the mechanical stability of the coating. Few conductive hydrogel (CH) systems have been developed for neural interface purposes(Kim et al., 2004, 2010; Green et al., 2012a, b; Hassarati et al., 2014), but preliminary studies have demonstrated the potential of this coating technology.

The CSC, impedance and charge injection limit of CH coated electrodes are significantly better than bare metal electrodes, but are also often improved compared to homogenous CPs (Cheung, 2007; Green et al., 2012b; Hassarati et al., 2014). Kim et al. (2004) electrodeposited PPy through a 1% alginate hydrogel and found that the resulting construct had an impedance of 7 kΩ at 1 kHz (for a 1257 μm^2^ electrode) which was approximately 14 times lower than the PPy films alone. In a similar study Chikar et al. (2012) deposited PEDOT through an alginate hydrogel and found that the CSC increased exponentially relative to the amount of PEDOT deposited within the hydrogel. This increase in CSC relative to the amount of PEDOT was more pronounced than that reported for PEDOT as a homogenous film, indicating that the same amount of material when deposited in a hydrogel had a greater impact on the electrical properties. It is generally thought that the hydrophilic mesh provided by the hydrogel promotes a large three-dimensional surface of conductive material which is available for charge transfer. This increase in surface area compared to the relatively dense and hydrophobic surface of a homogenous CP is expected to be the source of the enhanced electrochemical properties. The increase charge transfer area also leads to significant improvements in charge injection limit. In a study by Hassarati et al. (2014) a CH fabricated from PEDOT/PVA-Hep was applied to cochlear implants and increased the charge injection limit of the Pt electrodes by up to 24 times. This coating was also shown to be stable over 1 billion stimulations at clinically relevant levels.

**FIGURE 5:**
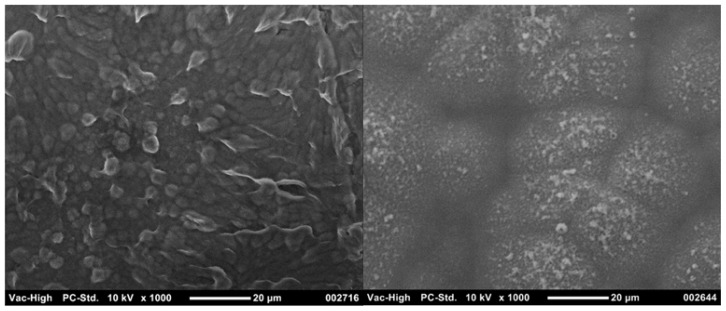
**Different morphologies of CHs can be produced through varying the hydrogel component.** PEDOT grown through 30 wt% heparin has a more cobblestone appearance (left) compared to the nodular PEDOT grown through 20 wt% PVA-Hep (right).

Several CH systems have been shown to reduce the stiffness of electrodes, and as expected the modulus of these hybrids typically lies between that of the component CP and hydrogel (Aouada et al., 2006; Baek et al., 2013b; Green et al., 2012b). Double network polymers of chemically synthesized PEDOT:PSS and polyacrylamide (PAAm) were found to have elastic moduli which ranged between 27 and 350 kPa. The increased stiffness was a function of both the percentage content of the stiffer PEDOT:PSS and the cross-link density of the PAAm (Aouada et al., 2006). When chemically synthesized CPs are used as a component of a CH the amount of CP can be carefully controlled, and has an impact on both the electrical conductivity and stiffness of the hybrid. However, when electrodeposited CPs are grown through hydrogels the elastic moduli of the resulting hybrid appears to be minimally variable (Green et al., 2012b). In a recent study, the stiffness of two different CHs was found to be close to 1 MPa, despite the CHs being fabricated from hydrogels with different chemistries and percentage of polymer content (Green et al., 2012b). The morphology of these CH variants was also dissimilar, as depicted in **Figure 5**, but both the electrical and mechanical properties were not significantly different. While there are several studies which report combining hydrogels with electrodeposited CPs for application to electrodes, few have quantified the modulus of these hybrids.

Conductive hydrogels have shown negligible *in vitro* cytotoxicity and good compatibility with several cell types (Green et al., 2010a; Mario Cheong et al., 2013). The biological properties of these composites reflect the properties of the component polymers, which have been shown to be compatible with a range of cell types. Specifically, CH materials have been used to support the attachment and growth of PC12 cells, the interleukin-3 dependent pro-B cell line (BaF3) and Schwann cells (Runge et al., 2010; Mario Cheong et al., 2013). *In vivo* PEDOT-alginate CHs (Kim et al., 2010) have been implanted in the auditory cortex area of the guinea pig. Despite the impedance of the hydrogel electrodes being much lower *in vitro*, the CH had similar impedance* in vivo* to that of uncoated electrodes. However, a significant improvement in SNR was found in the initial period following implantation. The impact of the material on the surrounding tissue was not examined histologically, but it was proposed that swelling of the construct increased the distance between the electrodes and target tissue (Kim et al., 2010). In a related field Abidian et al. (2012) developed PEDOT-agarose nerve conduits which were found to support nerve regeneration, although control autografts supported regeneration of significantly greater nerve fiber numbers. It was proposed that the PEDOT within the agarose inhibited cellular access to essential nutrients, limiting the development of functional nerve fascicles. It is also important to note that these studies did not utilize any active stimulation of tissue. It is clear that more information is required to successfully develop CHs and it is critical to assess the performance of these constructs *in vivo* under active implant conditions.

In preliminary studies it has been shown that CHs have the properties which can address the limitations of both metallic and CP coated electrodes by reducing mechanical mismatch, while maintaining improved electrical properties. Initial *in vitro* studies demonstrate the benefit of these coatings, but there are no studies which systematically compare the different types of hydrogels and CPs used to fabricate these materials. Furthermore, the integration of the two polymer systems is poorly understood, which limits the ability to manipulate the CH structure and tailor the system to display specific characteristics for neural interfacing. Optimization of CHs will require studies which examine the long-term performance of coated electrodes in a stimulated biological environment.

### BIOACTIVE COATINGS

All of the coating technologies discussed above can be modified to incorporate biological molecules. Cellular attachment molecules, anti-inflammatory agents and neurotrophins have been commonly investigated for improving the biological response of neural electrodes. Ideally, one of more of these molecules can be used to provide cues to bring neurons closer to electrodes and reduce the impact of the foreign body response. **Table 3** summarizes a range of biological molecules, which have been incorporated within or at electrode surfaces.

**Table 3:** Summary of the combinations of coatings and biomolecules which have been investigated for improving the interaction of electrodes and neural cells.

Application	Coating	Biomolecule	Reference
**Cell attachment**	PEDOT	DCDPGYIGSR and DEDEDYFQRYLI	Green et al. (2010b)
	PEDOT	DCDPGYIGSR and DEDEDYFQRYLI	Green et al. (2009)
	PEDOT	Fibronectin fragments DCDPGYIGSR	Cui and Martin (2003)
	PPy	Fibronectin fragments Nonapeptide CDPGYIGSR	Cui et al. (2001b)
	CNT	Collagen type IV	Nguyen-Vu et al. (2006)
**Anti-inflammatory**	PPy	HA	Collier et al. (2000)
	PPy	Dexamethasone	Wadhwa et al. (2006)
	PEDOT electrodeposited on PLGA nanofibers	Dexamethasone	Abidian et al. (2006)
	Polyterthiophene	Dexamethasone phosphate	Stevenson et al. (2010)
	Alginate hydrogel loaded with Poly(lactic-co-glicolide)(PLGA) nanoparticles	Dexamethasone	Kim and Martin (2006)
	Nitrocellulose	αMSH	Zhong and Bellamkonda (2005),^2007^
	Silicon	αMSH	He et al. (2007)
	Multiwalled CNTs	Dexamethasone	Luo et al. (2011a)
**Growth factors**	Methylcellulose	PDGF-BB and IGF-1	Wells et al. (1997)
	Poly (ethylene-co-vinyl acetate) (EVA) (EVA rods)	NGF, NT-3, BDNF and GDNF,	Bloch et al. (2001), Barras et al. (2002), Fine et al. (2002)
	Agarose	NGF and Laminin	Yu and Bellamkonda (2003)
	Collagen	NT-3	Houweling et al. (1998)
	PEG and PEG-PLA	NT-3	Burdick et al. (2006), Piantino et al. (2006)
	Agarose	BNDF	Jain et al. (2006)
	Fibrin	Heparin binding delivery system (HBDS) and NT-3	Taylor and Sakiyama-Elbert (2006)
	PPy	Laminin fragments RNIAEIIKDI (p20)	Stauffer and Cui (2006)
	PEG-PLA	NGF,	Winter et al. (2007)
	pHEMA-lysine	NGF,	Jhaveri et al. (2008)
	Fibrin	NGF	Lee et al. (2003)
	Multiwalled CNT	NGF	Matsumoto et al. (2007)

*
HA, hyaluronic acid; αMSH, α-melanocyte stimulating hormone; NGF, nerve growth factor; PDGF-BB, platelet derived growth factor-BB; IGF-1, insulin-like growth factor-1; BDNF, brain derived neurotrophic factor; GDNF, glial cell-derived neurotrophic factor; NT-3 – neurotrophin 3; HBDS, heparin binding delivery system; PEG, poly(ethylene glycol); PEG-PLA, poly(ethylene glycol)-poly(lactic acid); and CNT, carbon nanotube.
*

The provision of cell attachment molecules within coatings has been extensively explored. In studies which aim to restoring neuronal function, it has been proposed that extracellular matrix (ECM) molecules can provide structural and chemical cues to encourage regeneration at the neural interface (Chan and Mooney, 2008). ECM molecules such as collagen, laminin, and fibronectin among others, have been regarded as key elements in cellular attachment, cell growth, homeostasis, differentiation and cellular motility (Gumbiner, 1996; Banker and Goslin, 1998). Each molecule as well as related peptide sequences can provide mechanical, biological and chemical cues for neural tissue growth and development. Comprehensive reviews on the performance of specific proteins in the ECM have been previously published (Gumbiner, 1996; Banker and Goslin, 1998; Barros et al., 2011; Blau, 2013). **Table 3** lists some of the ECM molecules which have been included within synthetic polymers with an aim of imparting these cues at the device interface. The molecules are classified as proteins including laminin, fibronectin and collagen or related peptide sequences such as arginine–glycine-aspartic acid (RGD), polylysine and gelatin. Proteins and peptides can be incorporated within CNT, CP or CH coatings by simple adsorption, entrapment or covalent binding. It is known that adsorption does not produce a stable interface in the *in vivo* environment as competitive binding of the surrounding proteins can displace the adsorbed layer. Similarly, entrapped proteins can either diffuse through coatings, or if particularly large relative to the coating porosity, can be obstructed within the coating, such that they are not accessible to the cells. As a result cell attachment molecules are often covalently linked to either the coating surface or a component of the coating prior to fabrication. Such molecules have been bonded to CNTs or hydrogel macromer and alternatively used as a CP dopant ion (Cui et al., 2001b; Nguyen-Vu et al., 2007; Green et al., 2010b). Many studies have shown that these sequences encourage neural attachment, survival and differentiation *in vitro* (DeLong et al., 2005; Green et al., 2010b; Chikar et al., 2012). However, there is minimal evidence *in vivo* which supports the benefit of these components to the chronic neural interface. Cui et al. (2003) implanted PPy coated electrodes which were functionalized with laminin fragments in guinea pig cortex. It was shown that neuronal attachment was promoted on some of the electrodes but in spite of having good initial recordings, fibrotic tissues ultimately isolated the electrodes from the target tissue. While cell attachment proteins have clear benefit *in vitro*, there is a need to further explore their presentation and stability *in vivo*.

Anti-inflammatory molecules have been proposed for mitigating either the initial inflammatory reaction or the ongoing presence of fibrotic reactions at the neural interface. Unlike cell attachment proteins, anti-inflammatories must be delivered to the extracellular space to have an effect on the surrounding tissues. As a result these molecules are incorporated into coatings such that they can be delivered, either by passive diffusion or controlled delivery (Asplund et al., 2014). Dexamethasone is one of the most common drug molecules used to combat inflammation at the electrode interface, although hyaluronic acid (HA) and alpha-melanocyte-stimulating hormone (αMSH) have also been used. (Abidian et al., 2006) electrospun dexamethasone-loaded PLGA nanofibers, which were then encapsulated in PEDOT. In this highly conductive construct the dexamethasone could be delivered with a controlled dosage by electrical stimulation. However, due to the limited volume of the PEDOT coated nanofibres, the drug could only be delivered for 7 days at therapeutic levels. (Luo et al., 2011a) introduced dexamethasone to composite CNT-PPy coatings and also demonstrated release under electrical stimulation. The anti-inflammatory dose administered this way was enough to prevent microglia activation in a cell culture, but the ability to deliver therapeutic levels was restricted to a period of 24 h. In contrast (Zhong and Bellamkonda, 2005) entrapped α-MSH in nitrocellulose coatings which demonstrated slow, sustained release by diffusion over 21 days *in vitro*. The α-MSH was shown to retain bioactivity, and successfully inhibited microglial inflammatory responses. Nonetheless all of these approaches are limited as following depletion of the drug from the coating the desired low inflammation state was not maintained.

Inclusion of neurotrophins such as brain-derived neurotrophic factor (BDNF) (Richardson et al., 2007b) and nerve growth factor (NGF; Kim et al., 2007), within electrode coatings encourages the initial growth of neuronal processes towards implanted electrodes. This approach is designed to both initiate neural regeneration and also reduce the gap between the electrode and target tissue. However, several studies have shown that shrinkage of neurite growth occurs after the NGF supply is depleted (Fujita et al., 1989; Richardson et al., 2007a). While a substantial concentration of growth factor can be incorporated within hydrogels, there is only a very limited amount of any biological molecule which can be incorporated within CPs or CNTs (Richardson et al., 2007a). By removing the reliance on material loading and delivery rate (Gillespie et al., 2003) maintained spiral ganglion neurons for 28 days through BDNF administration via a mini-osmotic pump. It was expected that this method would provide a more controlled and longer treatment period than possible with coatings; however, an accelerated loss of neuronal survival occurred after cessation of the treatment. It is therefore expected that provision of neurotrophins at the neural interface will only be effective using a method which support chronic delivery. While current coating technologies do not meet this need, several tissue engineering constructs provide insight into approaches which may address this challenge.

While CNTs, CPs, and hydrogels can be functionalized with bioactive molecules to minimize inflammation and promote neuronal attachment, the main limitation is the ongoing administration of drugs at a therapeutic level. Additionally, at high concentrations it has been shown that these bulky molecules can severely impact on the coating physicochemical properties, in particular CP electrical and mechanical properties are diminished (Collier et al., 2000; Cui et al., 2001b; Green et al., 2009, 2010b). An advantage of CNTs is that their cylindrical morphology can been used as a reservoir for bioactive agents, however they require time intensive functionalization processes in order to be soluble and dispersed in common solvents and aqueous drug solutions (Georgakilas et al., 2002; Bianco et al., 2005).

Current coating technologies including CNTs, CPs, hydrogels, and CHs address several of the limitations of metallic electrodes used in neural interfacing. However, these materials by themselves do not provide a platform for chronic biochemical support which can prevent fibrotic tissue growth and encourage neural cell interactions. In the field of tissue engineering it has been shown that biochemical support (Bhatheja and Field, 2006; Allen and Barres, 2009). can be maintained through the provision of encapsulated cells (Johnson et al., 1989; Majumdar et al., 2011; Daud et al., 2012; Higashimori and Yang, 2012) which naturally produce a range of growth factors and extracellular matrix proteins (Bhatheja and Field, 2006; Allen and Barres, 2009).

A more complex, but highly functional interface will utilize coating technologies in combination with tissue engineered cellular constructs.

## TISSUE ENGINEERING THE NEURAL INTERFACE

Ideally, materials used at the neural interface must both minimize the foreign body response as well as promote neuronal survival to maintain the target neurons in close proximity to the electrodes. A more recent idea which has evolved from the literature is the fabrication of electrodes which are intimately associated with neural tissue (Wilson and Dorman, 2008; Richter et al., 2011; Green et al., 2013a). This approach combines the principles of tissue engineering with neuroprosthetic electrode coatings and proposes the incorporation of neural cells within the device itself. Ultimately, polymer systems can be modified to not only improve the electrode properties, but also support the encapsulation and survival of neural cells.

The concept of integrating living cells with electrodes was first raised in 1980, when Ochiai et al. proposed using live blue-green algae embedded in an alginate gel to act as a solar to electric energy photoconverter (Ochiai et al., 1980). They coined the term, “living electrode.” In a follow up study, (Ochiai et al., 1983) noted that although stability of these biological electrodes was good, the power conversion efficiency was very low at approximately 0.1%. The latter limitation in efficiency restricted further development of this concept. In 1999, non-nucleated cells were successfully embedded in the PPy electrodeposited onto metal electrodes (Campbell et al., 1999). Cell surface antigen integrity was maintained and because the biosensor application required only presentation of cell surface antigens, there was no requirement for long-term viability and cell function. More recently, Richardson-Burns et al. described electrodeposition of PEDOT on neuroblastoma-derived cell lines and primary mouse cortical cells grown on metal electrodes (Richardson-Burns et al., 2007). Cells survived initial short-term exposure to monomers, but apoptosis was observed at 72 h with up to a third of the cells dying by 120 h. Cytoskeletal disruption was noted soon after polymerization of PEDOT suggesting that focal adhesions may have been disrupted (Richardson-Burns et al., 2007). Despite subsequent research from the same group (Sarah et al., 2007; Ouyang et al., 2011), there has been little progress over the past 5 years on the concept of creating “living” electrodes that contain eukaryotic cells.

**FIGURE 6:**
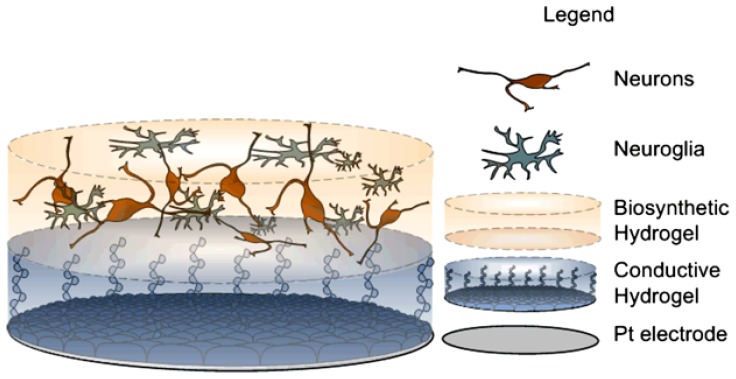
**Scheme of proposed construct, derived from Green et al. (2013a) bottom, Pt electrode site. Middle, conductive hydrogel coating and top, neural network encapsulated within degradable hydrogel**.

Pioneering studies by Richter et al. (2011) present polymeric devices seeded with stem cells. In this study, fibrin hydrogel was used to protect cells from shearing stress similar to that experienced upon neural electrode implantation. More recent reports have shown a proof of concept of the feasibility of building layered constructs of cell loaded hydrogels over CP coated Pt electrodes (Green et al., 2013a). This layered construct involves a hydrogel scaffold to hold neuronal cells while maintaining electrical characteristics of metallic electrodes coated with PEDOT. The concept behind this layered construct is that if neuroprogenitor cells can be supported and developed such that neurite processes are directed towards the target tissue, as depicted in **Figure 6**, then the electrode will be surrounded by a functional neural layer instead of fibrotic tissue. Several challenges arise in the fabrication of such a construct. Importantly, the hydrogel scaffold must be tailored to provide mechanical and biochemical properties that support the growth and differentiation of neural cells. Additionally, neurite outgrowth should be promoted in a vectorial fashion towards the target tissue. Finally, the encapsulated cells must form functional synaptic processes with the target neurons. These issues present great challenges, however, it is expected that recent research in tissue engineering will provide insights to guide development of such cellular constructs (Schmidt et al., 1997; Guimard et al., 2007; Sidhu et al., 2012). For example, it has been shown that cell survival and differentiation in three dimensional scaffolds is greatly improved by seeding cells with complementary accessory cells which would be normally present in the developing tissue (Sidhu et al., 2012). These cells generate a variety of biochemical cues producing ECM proteins and growth factors which support the attachment, survival, and differentiation of the functional cells within the scaffold. As such functional living electrodes are likely to require not only neural cells but also supporting neuroglia to achieve functional networks with target tissue.

Despite the substantial amount of research required to develop this technology, it has the potential to provide a pathway for maintaining neurons in close proximity with electrodes. Additionally, a fully realized tissue engineered electrode may prevent scar tissue encapsulation and provide a means of communicating with diseased or damaged cells using natural synaptic processes. Embedded neurons can provide single-cell electrodes which address individual target cells and this innovative method of communicating across the neural interface has the capacity to increase the spatial resolution of bionic devices.

## CONCLUDING REMARKS

Carbon nanotubes, CPs, and hydrogels provide potential materials to enhance electrical performance of microelectrodes by increasing electrochemical area. However, to develop a long term neural interface it is critical to determine the interplay of the mechanical, electrical and biological properties. The true potential of these materials on neural interfaces is not completely understood as the many studies have been limited to characterization in the *in vitro* environment. An important component of future research is the assessment of organic coatings *in vivo* under chronic implant conditions. Systematic studies are required to assess the different coating technologies and provide direction on which material or combination of materials will provide the most beneficial characteristics for neural interfacing. Tissue engineered living electrodes may provide an alternate technology platform which will enable the development of a truly innovative way of communicating with the neural system through synaptic processes.

## Conflict of Interest Statement

The authors declare that the research was conducted in the absence of any commercial or financial relationships that could be construed as a potential conflict of interest.
